# Clinicians' awareness of the Affordable Care Act mandate to provide comprehensive tobacco cessation treatment for pregnant women covered by Medicaid

**DOI:** 10.1016/j.pmedr.2015.08.013

**Published:** 2015-08-22

**Authors:** Van T. Tong, Lucinda J. England, Ann Malarcher, Jeanne Mahoney, Britta Anderson, Jay Schulkin

**Affiliations:** a Division of Reproductive Health, Centers for Disease Control and Prevention, Atlanta, GA, USA; b Office on Smoking and Health, Centers for Disease Control and Prevention, Atlanta, GA, USA; c American College of Obstetricians and Gynecologists, Washington, DC, USA

**Keywords:** Smoking cessation, Pregnancy, Medicaid, Insurance, Obstetricians–gynecologists

## Abstract

The Affordable Care Act (ACA) requires states to provide tobacco-cessation services without cost-sharing for pregnant traditional Medicaid-beneficiaries effective October 2010. It is unknown the extent to which obstetricians–gynecologists are aware of the Medicaid tobacco-cessation benefit. We sought to examine the awareness of the Medicaid tobacco-cessation benefit in a national sample of obstetricians–gynecologists and assessed whether reimbursement would influence their tobacco cessation practice. In 2012, a survey was administered to a national stratified-random sample of obstetricians–gynecologists (n = 252) regarding awareness of the Medicaid tobacco-cessation benefit. Results were stratified by the percentage of pregnant Medicaid patients. Chi-squared tests (p < 0.05) were used to assess significant associations. Analyses were conducted in 2014. Eighty-three percent of respondents were unaware of the benefit. Lack of awareness increased as the percentage of pregnant Medicaid patients in their practices decreased (range = 71.9%–96.8%; *P* = 0.02). One-third (36.1%) of respondents serving pregnant Medicaid patients reported that reimbursement would influence them to increase their cessation services. Four out of five obstetricians–gynecologists surveyed in 2012 were unaware of the ACA provision that required states to provide tobacco cessation coverage for pregnant traditional Medicaid beneficiaries as of October 2010. Broad promotion of the Medicaid tobacco-cessation benefit could reduce treatment barriers.

## Highlights

• Medicaid provides no cost tobacco-cessation services for pregnant patients.
• We surveyed obstetricians–gynecologists' awareness of the tobacco-cessation benefit.
• Majority (83%) of obstetricians–gynecologists were unaware of the benefit.
• One-third may increase cessation services because of reimbursement.
• Broad promotion of the Medicaid tobacco-cessation benefit is needed.

## Introduction

Tobacco use during pregnancy is the most common cause of preventable poor infant outcomes (e.g., preterm delivery, low birth weight) for which effective interventions exist (Chamberlain et al., 2013, U.S. Department of Health Human Services, 2014). In addition, prenatal smoking is associated with an estimated $122 million in excess infant health care costs at delivery in the United States (Adams et al., 2011). Significant disparities exist between low and high socioeconomic status women, particularly among women enrolled in Medicaid. Smoking prevalence during and after pregnancy was 17.6% and 23.4%, respectively, among Medicaid enrolled women versus 5.2% and 9.3% among privately insured women (Tong et al., 2013). Considering that Medicaid is the largest payer of prenatal and delivery healthcare and covers 45% of US births (The Henry J. Kaiser Family Foundation, 2014), the potential cost-savings of eliminating tobacco use and averting poor birth outcomes in the pregnant Medicaid population could be substantial (Adams et al., 2011, Lightwood et al., 1999).

The Affordable Care Act (ACA) requires states to provide tobacco-cessation services, including counseling and pharmacotherapy, without cost-sharing (i.e., no out-of-pocket costs) for pregnant traditional Medicaid beneficiaries effective October 2010 (USDHHS, 2011). However, it is unknown the extent to which obstetricians–gynecologists are aware of the Medicaid tobacco-cessation benefit. We examined awareness of the Medicaid tobacco-cessation benefit in a national sample of obstetricians–gynecologists and assessed whether reimbursement would influence their cessation practice. These findings can be useful to inform state maternal and child health and tobacco control efforts to reduce prenatal smoking.

## Methods

During February–August 2012, the American College of Obstetricians and Gynecologists (ACOG) conducted a mailed survey of a national stratified-random sample of practicing obstetricians–gynecologists. Detailed methodology has been described previously (Coleman-Cowger et al., 2014). Briefly, 425 Collaborative Ambulatory Research Network (CARN) and 599 non-CARN members were invited to participate. CARN members are clinicians who volunteer to participate in ACOG surveys. Those invited received an introductory letter and up to 3 reminders. Response rates were 52% (CARN) and 31% (non-CARN). The sample was further restricted to clinicians providing obstetrical care (n = 252, 62% of respondents). The study was deemed exempt from review by ACOG and the Centers for Disease Control and Prevention Institutional Review Boards.

The survey focused on practice patterns and opinions related to patient tobacco use. The two survey questions analyzed for this study were: 1) “Are you aware that the ACA includes a provision that requires that pregnant women on Medicaid receive coverage for comprehensive smoking cessation services, including both counseling and pharmacotherapy?”; 2) “How much influence would reimbursement for cessation services for pregnant women on Medicaid under the ACA have on how you provide cessation services?” Results were stratified by the categorical response of the percentage of pregnant Medicaid patients seen by respondents (0, 1–24%, 25–50%, > 50%). Chi-squared tests (p < 0.05) were used to assess significant associations. Analyses were conducted in 2014.

## Results

The majority of respondents were female (55.8%) and non-Hispanic White (84.3%); on average, respondents completed residency 19 years ago. Most respondents practiced in urban/suburban locations (81.0%), and 30.6% provided comprehensive primary care for women. About a quarter of respondents had > 50% pregnant Medicaid patients; 61.6% had < 50% pregnant Medicaid patients; and 13.2% had no pregnant Medicaid patients (Table 1).

Overall, 83% of obstetricians–gynecologists were unaware of the Medicaid tobacco-cessation benefit for pregnant patients. Lack of awareness increased as the percentage of pregnant Medicaid patients in their practices decreased (range = 71.9%–96.8%; *P* = 0.02) (Table 1). Of respondents who saw pregnant Medicaid patients, one-third (36.1%) said reimbursement would increase their cessation services, and nearly 40% of those with > 50% Medicaid patients said they would increase their services. A substantial fraction (30.2%) of respondents reported that cessation services would not change because reimbursement wouldn't address ‘existing barriers to delivering service’, and 16.2% said they did not know how reimbursement would affect their cessation practices.

## Discussion

We found that 4 out of 5 obstetricians–gynecologists surveyed in 2012 were unaware of the ACA provision that required states to provide tobacco cessation coverage for pregnant traditional Medicaid beneficiaries. However, one-third of respondents reported that reimbursement would influence them to increase cessation services, and an even greater percentage was seen among respondents who saw more Medicaid-enrolled patients. A previous study suggests that states with more comprehensive Medicaid coverage of tobacco cessation treatments, primarily through coverage of medications, resulted in 1.6 percentage point reduction (p < .05) in smoking before pregnancy and a small increase (< 1 day) in infant gestation (Adams et al., 2013). In addition, as counseling will also be covered by the ACA mandate, a meta-analysis of 77 trials found that psychosocial interventions are effective in increasing the proportion of women who stop smoking in late pregnancy, and women who received psychosocial interventions had an 18% reduction in preterm births and infants born low birth weight (Chamberlain et al., 2013). Hence, reducing barriers to cessation treatments, as such through a comprehensive tobacco cessation benefit, could potentially allow more smokers to access treatment, increase cessation and improve infant outcomes among pregnant Medicaid enrollees.

Comprehensive and well-publicized benefits have shown larger effects in quitting among the general population of Medicaid-enrollees in the state of Massachusetts, including among young people and women (Land et al., 2010). For providers, this promotion included the development of fact sheets, with rate and billing codes, a pharmacotherapy pocket guide, and new intake and assessment protocols that were widely disseminated to health care systems and facilities (CDC, 2014). In addition, the state also directed educational campaigns to consumers, tracked the use of the benefits, and provided feedback and recognition to providers who were regularly referring patients. Acknowledging the importance of raising awareness of the tobacco cessation benefit, the Centers for Medicare and Medicaid Services placed information on their website (Centers for Medicare and Medicaid Services, 2014) about Medicaid tobacco-cessation benefits for pregnant women and non-pregnant enrollees (Singleterry et al., 2014). However, broad state promotion and outreach of the Medicaid tobacco-cessation benefits, as noted earlier, for pregnant women can help to increase treatment utilization.

A substantial percentage of respondents reported that reimbursement would be insufficient to address existing barriers for cessation. Provider barriers that have been reported in a previous analysis of this data included time limitations to deliver cessation services in prenatal care visits and patient's resistance to intervention (Coleman-Cowger et al., 2014). While reimbursement may improve service provision and broad promotion of the benefits may increase awareness, additional strategies, such as provider training (Tong et al., 2012) and healthcare system changes to facilitate stream-lined screening and treatment (Fiore et al., 2008), are also important to increase treatment utilization. Educational campaigns directed to consumers could also stress the importance and/or benefits of quitting smoking and support that prenatal care staff can provide.

This study has limitations to note. First, the study is limited by the low survey response rates (31–52%), which is consistent with previous ACOG surveys. However, nonresponse bias has been shown to be minimal among physician groups compared to other groups (Kellerman and Herold, 2001). Second, the sample size is small. For our analysis of how reimbursement would influence cessation services, we had limited power to test for differences in whether reimbursement would influence cessation services by percentage of Medicaid patients seen. Finally, these data are self-reported, and we did not verify information regarding awareness with their actual cessation or billing practices.

In conclusion, four out of five obstetricians–gynecologists surveyed in 2012 were unaware of the ACA provision that required states to provide tobacco cessation coverage for pregnant traditional Medicaid beneficiaries, and a third of respondents serving pregnant Medicaid patients reported that reimbursement would influence them to increase their cessation services. Promoting awareness of the Medicaid tobacco-cessation benefit among all medical providers who see pregnant and reproductive-aged women could help to reduce treatment barriers, thereby increasing cessation and improving maternal and infant health.

## Conflicts of interest

The authors report no conflict of interest.

## Figures and Tables

**Table 1:** Obstetricians–gynecologists' awareness of Medicaid tobacco-cessation benefit for pregnant patients covered by Medicaid (n = 252).

	Total %	Percent of pregnant patients covered by Medicaid				
0%	1–24%	25–50%	> 50%	p-Value^a^
*Total*	100.0%	13.2%	30.2%	31.4%	25.2%	
*Awareness of Medicaid tobacco-cessation benefit for pregnant women*						
Yes	17.0%	3.2%	15.3%	16.0%	28.1%	0.02
No	83.0%	96.8%	84.7%	84.0%	71.9%	
*Will ACA provision influence reimbursement for cessation services?*^b^						
It won't change how I provide services because our services are already adequate	7.4%	NA	15.5%	1.4%	5.2%	0.09
It won't change how I provide services as it won't address existing barriers to delivering service	30.2%	NA	29.6%	32.9%	27.6%	
It won't change for other reasons	5.4%	NA	5.6%	2.7%	8.6%	
I will likely increase our services because of improvement in reimbursement	36.1%	NA	31.0%	38.4%	39.7%	
I will likely increase our services for other reasons	4.5%	NA	1.4%	6.8%	5.2%	
I don't know	16.3%	NA	16.9%	17.8%	13.8%	

ACA = Affordable Care Act.

a Based on chi-square tests.

b Respondents (n = 202) could only mark one response.
